# The Homozygote VCP^R155H/R155H^ Mouse Model Exhibits Accelerated Human VCP-Associated Disease Pathology

**DOI:** 10.1371/journal.pone.0046308

**Published:** 2012-09-28

**Authors:** Angèle Nalbandian, Katrina J. Llewellyn, Masashi Kitazawa, Hong Z. Yin, Mallikarjun Badadani, Negar Khanlou, Robert Edwards, Christopher Nguyen, Jogeshwar Mukherjee, Tahseen Mozaffar, Giles Watts, John Weiss, Virginia E. Kimonis

**Affiliations:** 1 Department of Pediatrics, University of California Irvine, Irvine, California, United States of America; 2 Department of Molecular and Cell Biology, University of California Merced, Merced, California, United States of America; 3 Department of Neurology, Anatomy and Neurobiology, University of California Irvine, Irvine, California, United States of America; 4 Department of Pathology and Lab Medicine, University of California Los Angeles, Los Angeles, California, United States of America; 5 Department of Pathology, University of California Irvine, Irvine, California, United States of America; 6 Department of Radiological Sciences, University of California Irvine, Irvine, California, United States of America; 7 Department of Orthopedics, University of California Irvine, Irvine, California, United States of America; 8 Department of Cell Biology and Biochemistry, University of East Anglia, Norwich, Norfolk, United Kingdom; Brigham and Women's Hospital, Harvard Medical School, United States of America

## Abstract

Valosin containing protein (VCP) mutations are the cause of hereditary inclusion body myopathy, Paget's disease of bone, frontotemporal dementia (IBMPFD). *VCP* gene mutations have also been linked to 2% of isolated familial amyotrophic lateral sclerosis (ALS). VCP is at the intersection of disrupted ubiquitin proteasome and autophagy pathways, mechanisms responsible for the intracellular protein degradation and abnormal pathology seen in muscle, brain and spinal cord. We have developed the homozygous knock-in VCP mouse (VCP^R155H/R155H^) model carrying the common R155H mutations, which develops many clinical features typical of the VCP-associated human diseases. Homozygote VCP^R155H/R155H^ mice typically survive less than 21 days, exhibit weakness and myopathic changes on EMG. MicroCT imaging of the bones reveal non-symmetrical radiolucencies of the proximal tibiae and bone, highly suggestive of PDB. The VCP^R155H/R155H^ mice manifest prominent muscle, heart, brain and spinal cord pathology, including striking mitochondrial abnormalities, in addition to disrupted autophagy and ubiquitin pathologies. The VCP^R155H/R155H^ homozygous mouse thus represents an accelerated model of VCP disease and can be utilized to elucidate the intricate molecular mechanisms involved in the pathogenesis of VCP-associated neurodegenerative diseases and for the development of novel therapeutic strategies.

## Introduction

Hereditary inclusion body myopathy (hIBM), caused by mutations in the *VCP* gene, is associated with weakness and atrophy of skeletal, pelvic and shoulder girdle muscles in 90%, Paget disease of bone (PDB) in 50%, and frontotemporal dementia (FTD) in approximately 30% of individuals (as reviewed in [1]) [2], [3], [4]. Patients exhibit scapular winging and progressive muscle weakness and ultimately die from cardiac and respiratory failure, typically in their 50's to early 60's [2], [5]. Histologically, patients display rimmed vacuoles and trans-activator regulatory DNA binding protein-43 (TDP-43)-positive ubiquitinated inclusion bodies in muscles [2], [5], [6], [7]. Patients are often diagnosed with limb girdle muscular dystrophy (LGMD), and approximately 15% manifest classic amyotrophic lateral sclerosis (ALS) [5], [8], [9], a rapidly progressing neurodegenerative disease involving both upper (UMNs) and lower (LMNs) motor neurons [10]. Approximately 10% of ALS is familial (fALS), associated with mutations in copper/zinc superoxide-dismutase-1 gene (SOD1), fused in sarcoma/translocated in liposarcoma gene (FUS/TLS), TDP-43, valosin containing protein (VCP) and a hexanucleotide expansion of the C9orf 72 (chromosome 9 open reading frame 72) gene. *VCP* is associated with 1–2% of fALS [11] and has been recently identified in sALS [12]. Understanding the underlying molecular mechanisms by which mutations in *VCP* cause motor neuron degeneration, thus, will provide a unique opportunity of understanding the mechanism of the more common types of ALS and novel treatments.

VCP, a member of the type II AAA (ATPases associated with diverse cellular activities) family possesses two ATPase domains [13] and plays a critical role in a broad range of cellular activities, such as homotypic membrane assembly, endoplasmic reticulum-associated degradation of proteins (ERAD), the ubiquitin-proteasome system (UPS), cell cycle regulation, DNA repair, prevention of polyglutamine aggregation, autophagosome maturation in autophagy and mitophagy [14], [15]. Mutations in *VCP* are primarily in the N-terminal domain involved in ubiquitin binding and protein-protein interactions [6], [16], however mutations in other domains have also been identified. VCP-associated disease is increasingly being recognized worldwide, with 26 mutations having been identified thus far in families from Germany [17], [18], France [19], Austria [20], Italy [21], [22], UK [23], Australia [24], Brazil [25], Korea [26], [27] and the US [28], [29]. The R155H mutation is by far the most common, accounting for approximately 50% of affected individuals [1], [6], [30]. VCP is important in mediating protein degradation, a highly significant process for terminally differentiated cells. VCP is also important for the retro-translocation of mis-folded endoplasmic reticulum (ER) proteins, and failure in this activity results in defective ERAD and ER stress responses [31], [32].

Heterozygote VCP knock-in mouse models containing the common R155H *VCP* mutation were generated in our laboratory to study the effects of this *VCP* mutation *in vivo* [33]. Both human and mouse VCP proteins consist of 806 amino acids, and the mouse protein differs by only one amino acid residue (at position 684) when compared to the human protein containing the common R155H VCP human disease mutation. The heterozygote R155H mice demonstrated progressive muscle, bone, brain and spinal cord pathology when compared with their wild-type (WT) littermates [34], [35]. Although the heterozygote mice recapitulate the disease observed in patients with VCP mutations, the onset of the disease is relatively slow with pathology beginning around 6–9 months of age. Thus, we generated the R155H homozygous (VCP^R155H/R155H^) mice to investigate pathogenesis and monitor the response to therapeutic strategies more rapidly. These homozygote mice are small and denuded, demonstrate stunted growth and are weak with abnormal skeletal muscle architecture. They die by 14–21 days from accelerated muscle, spinal cord and cardiac pathology. Muscle histology reveals variability in muscle fiber size, centrally localized nuclei, and fiber necrosis. The VCP^R155H/R155H^ homozygote animals have prominent myopathic changes on EMG. Electron microscopy (EM) reveals abnormal mitochondrial structures in the muscle, brain and heart of these homozygote mice. Immunohistochemical studies show that the VCP^R155H/R155H^ mice develop prominent ubiquitin-positive aggregates, TDP-43 inclusions and abnormal autophagy pathology.

In order to develop treatments, it is necessary to understand the pathological molecular cascades that result in the clinical manifestation of the disease. Therefore, this mouse model provides a platform for developing such novel therapeutic strategies to treat VCP-associated and related neurodegenerative diseases.

## Materials and Methods

### Ethics Statement

All experiments were performed with the approval of the Institutional Animal Care and Use Committee (IACUC) at University of California, Irvine (UCI) (IACUC Protocol #2007-2716-1), and in strict accordance with the guidelines established by the National Institutes of Health (NIH). Animals were housed in the animal facility and maintained under constant temperature (22°C) and humidity, with a controlled 12∶12-h light-dark cycle. Mice were provided standard rodent chow (Harlan Teklad Rodent Diet, #8604, Madison, WI) and water *ad libitum*. Weight measurements and survival of the VCP^R155H/R155H^ and WT animals was monitored on a weekly basis. Mice were euthanized by CO_2_ inhalation followed by cervical dislocation or by cardiac perfusion in efforts to minimize suffering.

### Electron Microscopy

Quadricep muscles, hearts, and brains from WT and VCP^R155H/R155H^ littermates were fixed in 4% paraformaldehyde/0.1% glutaraldehyde in 0.1 M PBS for 24 h at 4°C. Tissue samples were fixed in 1% Osmium for 1 hr at 4°C and serially dehydrated in ethanol. Muscle samples were embedded in Eponate 12 resin at 65°C for 24–36 h. Ultrathin (60∼80 nm) sections were cut with a diamond knife. Sections were stained in 1% uranyl acetate for 30 min at room temperature, followed by lead citrate incubation for 7–10 min at room temperature. Electron micrographs were taken with a Gatan UltraScan US1000 digital camera and analyzed for architectural differences and lipid accumulation.

### Immunohistochemical Analyses

Cardiac perfusion was used for immunohistochemical characterization of the pathology of the VCP^R155H/R155H^ and WT animals. Mice were anesthetized with Ketamine and perfused transcardially with phosphate buffered saline (PBS), followed by 4% paraformaldehyde (PFA) for 10 minutes, after which brains were dissected. Tissues were subsequently incubated in 30% sucrose/PBS gradients for another 2 days and later processed for immunohistochemical analyses.

Quadricep muscles and brains from 21-day old VCP^R155H/R155H^ and WT were harvested and embedded in cryo-sectioning mounting media (Electron Microscopy Sciences, Hatfield, PA, USA) and stored at −80°C before sectioning (5–10 µm). For immunohistochemical analyses, sections were stained with TDP-43 (Abcam, Cambridge, MA, USA), ubiquitin (Abcam), VCP (Thermo Scientific, Waltham, MA, USA), LC3-I/II (Abcam), p62/SQSTM1 (Abcam), and COX-IV (Abcam) primary antibodies. Subsequently, sections were washed with 1× PBS and incubated with fluorescein-conjugated secondary antibodies (Sigma-Aldrich, St. Louis, MO, USA) for 1 hour at room temperature and mounted with DAPI-containing mounting media (Vector Laboratories, Inc., Burlingame, CA, USA) or TOTO-3 (Invitrogen Life Technologies, Inc., Carlsbad, CA, USA) and analyzed by fluorescence microscopy. Additionally, Hematoxylin and Eosin (H&E) staining was performed using routine methods and analyzed by light microscopy (Carl Zeiss, Thornwood, NY, USA).

### Staining of Mitochondrial Markers

Histochemical analyses and activity levels with Succinic Dehydrogenase (SDH) and Nicotinamide Adenine Dinucleotide (NADH) were performed with quadriceps muscle from the WT and VCP^R155H/R155H^ mice as previously described [36]. Briefly, quadricep muscle cross-sections were incubated with SDH (Sigma-Aldrich), NADH for 2 hours in the incubator at 37°C. Following incubation, slides were cooled for 5 minutes at room temperature and transferred to distilled water. Slides were then mounted with Aquamount (Thermo Scientific). The staining intensity was evaluated using a light microscope using an AxioVision image capture system (Carl Zeiss).

Neutral lipids in quadricep tissue sections from the VCP^R155H/R155H^ and WT mice were determined by Oil Red O staining. Briefly, frozen muscle sections were fixed in 10% formaldehyde for 1 minute and washed in tap water. Sections were stained with fresh Oil Red O for 10 minutes and subsequently washed in tap water. Next, sections were stained for 1 minute in Harris Modified Hematoxylin solution with acetic acid (Fisher Scientific, Pittsburgh, PA, USA) and washed briefly. Subsequently, sections were placed in bluing solution, washed in tap water, and mounted with Aquamount (Thermo Scientific). Staining intensity (fat, intense red; nuclei, blue) was evaluated using light microscopy (Carl Zeiss).

### Western Blotting Analyses

Quadricep muscle samples from 21-day old VCP^R155H/R155H^ and WT were harvested and protein was extracted using the NE-PER Nuclear and Cytoplasmic Extraction Kit (Thermo Scientific). Protein concentrations were determined using the Nanodrop according to the manufacturer's protocols. Equal amount of proteins were separated on Bis-Tris 4–12% NuPAGE gels (Invitrogen Life Technologies Inc.), and the expression levels of proteins were analyzed by Western blotting using VCP (Thermo Scientific), TDP-43 (Abcam), p62/SQSTM1 (Sigma-Aldrich), LC3-I/II (Abcam), COX-IV (Abcam) and ubiquitin-specific (Abcam) antibodies. Equal protein loading was confirmed by α tubulin, β actin, or GAPDH control antibodies (Santa Cruz Biotechnology, Santa Cruz, CA, USA) staining.

### MicroCT Imaging

MicroCT scan was performed by scanning the WT and VCP^R155H/R155H^ animals with a large area CT camera (30–40 micron high resolution, low noise, 14-bit x-ray imaging detector with 4096×4096 pixels). The high performance 64-bit work station controls the Inveon multimodality scanners. The reconstructed microCT images were analyzed and 3D trabecular structural parameters were determined using the Inveon Multimodality 3D Visualization software.

### Electromyography (EMG) Studies

Neurophysiological recordings in the limbs of WT and VCP^R155H/R155H^ mice were performed *in vivo* under Ketamine/Xylazine anesthesia. Terminal electromyography (EMG) study was performed on these animals at an average age of 14–21 days old. Subdermal EEG electrodes were used for conducting EMG studies; the active and reference electrodes were paired together to act as a concentric needle electrode for these recordings. The following muscles were sampled in these mice: bilateral tibialis anterior, bilateral hamstrings and bilateral medial gastrocnemius muscles as well as unilateral thoracic paraspinal muscles. All recordings were made using a Cadwell Sierra LT machine (Cadwell Laboratories, Kennewich, WA, USA). Patterns of insertional and spontaneous activity were noted in all these muscles along with pattern of motor unit potentials evoked by movement of limbs caused by noxious stimuli given to the footpads.

### Statistical Significance

Means were used as summary statistics for all experiments. We compared the weights, activity, EMG, Westerns and IHC studies among VCP^R155H/R155H^ (n = 10) and WT (n = 10) mice using mixed model analysis of variance and tested at P<0.05 levels using a Student's *t*-test assuming unequal variances. Histological analyses for the brains, hearts, and muscles characteristics derived from EM were compared between VCP^R155H/R155H^ (n = 10) and WT (n = 10) mice (ages 7–14 days) using Fisher's exact test with a one-sided 5% significance value and 80% power to detect a difference of 60% in frequency of histological and structural characteristics (sample size of 10 per group).

## Results

### Homozygote VCP^R155H/R155H^ model displays growth retardation and early lethality

**Figure 1:**
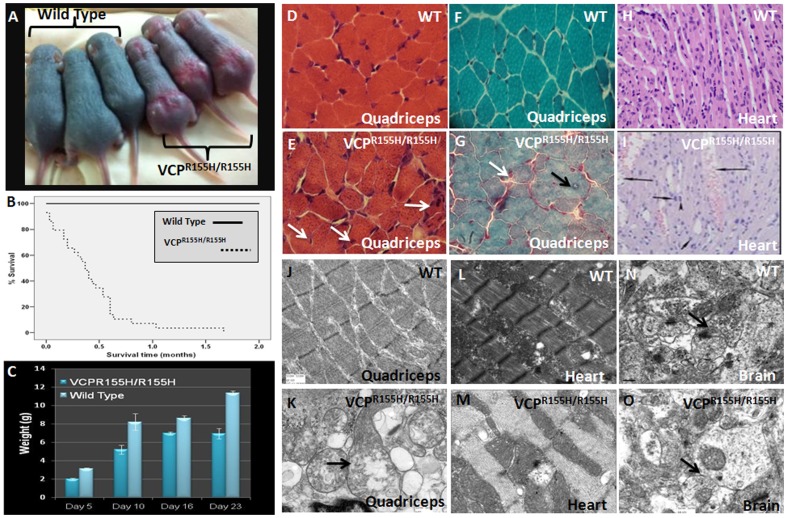
Weight and histopathological analyses of WT and homozygous VCP^R155H/R155H^ mice. (A) VCP^R155H/R155H^ mice are much smaller and weaker and demonstrate gross abnormalities such as hairless areas with ichthyosis-like thickening of the superficial layers of the skin compared to their WT littermates at day 14. MicroCT showed kyphosis of the homozygote mice. (B) Kaplan-Meier survival curve of WT and VCP^R155H/R155H^ mice. (C) Histogram demonstrating significant weight differences of WT and VCP^R155H/R155H^ mice. H&E images of (D) WT and (E) VCP^R155H/R155H^ mice demonstrating muscle pathology (Mag 63×). There was significant decrease in muscle mass with wide variation in muscle fiber size, central nuclei, split fibers and extensive fiber necrosis. Trichrome Gomori staining in (F) WT and (G) VCP^R155H/R155H^ mice revealed prominent staining in the periphery of the muscle fibers (Mag 63×). Histological H&E heart images of (H) WT (Mag 3200×) and (I) VCP^R155H/R155H^ mice (Mag 11000×) showing capillary dilations. EM analyses of quadriceps in (J) WT showing normal sarcomeric architecture (Mag 3200×) and (K) VCP^R155H/R155H^ mice revealing mitochondrial proliferation and architecture with megaconia and disrupted cristae (arrow) (Mag 11000×). EM analyses in (L) WT mice depicting normal heart histology and (M) VCP^R155H/R155H^ revealing enlarged vacuoles, highly disorganized and dilated vascular channels. EM analyses in (N) WT mice demonstrating normal brain histology (Mag 11000×) and (O) VCP^R155H/R155H^ revealing absence of well-defined synaptic complexes and post-synaptic enlargement in addition to vesicular degeneration (Mag 11000×).

We discovered early lethality in the VCP^R155H/R155H^ since litters from the heterozygote matings produced fewer homozygote mice than the expected 25% Mendelian ratio. These mice were smaller at birth and remained proportionally small, exhibited muscle weakness displaying poor mobility compared to their WT and heterozygote littermates. The mice had hairless patches of skin resembling ichthyosis-like thickening (**Figure 1A**). Early lethality of the homozygote animals ranged from birth to 21 days of age as analyzed by the Kaplan-Meier survival curve (**Figure 1B**). In contrast, the WT control and heterozygote littermates had a relatively normal lifespan [35]. The VCP^R155H/R155H^ mice had severe growth retardation and histological studies showed pathological abnormalities of the skeletal muscle fibers, heart and brain. The average weights for the WT and VCP^R155H/R155H^ animals were 3.13 g and 2.0 g at 5 days of age; 8.2 g and 5.2 g at 10 days of age; 8.62 g and 6.97 g at 16 days of age; and 11.35 g and 6.92 g at 23 days of age, respectively (p<0.05) (**Figure 1C**). There was no obvious skeletal dysplasia; however, the homozygote VCP^R155H/R155H^ mice displayed kyphosis on X-ray analysis.

### Severe muscle weakness and myopathic pathology in the homozygote VCP^R155H/R155H^ model

The VCP^R155H/R155H^ homozygote animals are significantly weaker and were not able to complete the grip strength test as compared to their WT littermates. In comparison to the WT mice (**Figure 1D**), the homozygote mice depicted a significant decrease in muscle mass, wide variation in fiber size, increased endomysial space, centrally localized nuclei and muscle fiber necrosis on quadriceps histology (**Figure 1E**). Compared to the WT mice which depicted normal histology (**Figure 1F**), trichrome staining revealed prominent mitochondrial staining and endomysial fibrosis in the VCP^R155H/R155H^ mice (**Figure 1G**). An increased number of internal nucleation and splitting myofibers in VCP^R155H/R155H^ animals were seen. Heart pathology by H&E depicted enlarged vacuoles, and dilated vascular channels in the VCP^R155H/R155H^ mice (**Figure 1I**) compared with the WT animals **(** **Figure 1H** **)**.

Electron microscopy (EM) of the WT quadriceps muscles demonstrated normal histology (**Figure 1J**), whereas the VCP^R155H/R155H^ mice quadriceps muscles revealed abnormal sarcomeric architecture and mitochondrial proliferation with large megaconia and disrupted cristae; with membrane bound material, possibly lipids and lysosomal structures with dense granules and glycogen content (**Figure 1K**). Compared to the WT littermates (**Figure 1L**), EM of the VCP^R155H/R155H^ heart revealed abnormal architecture of the channels and enlarged vacuoles (**Figure 1M**). Interestingly, echocardiograms of these VCP^R155H/R155H^ mice did not reveal any abnormalities in the left and right ventricle wall thickness and mass, chamber dilation or function (data not shown). We next evaluated the age-matched WT littermates and found normal architecture of the brain (**Figure 1N**), whereas the VCP^R155H/R155H^ brain depicted an absence of well-defined synaptic complexes, post-synaptic enlargement and vesicular degeneration (**Figure 1O**).

### Electromyography studies reveal myopathic changes in the VCP^R155H/R155H^ mouse

Electromyographic (EMG) examination of the VCP^R155H/R155H^ homozygote animals depicted prominent myopathic changes in the bilateral tibialis anterior, bilateral hamstrings and bilateral medial gastrocnemius muscles as well as unilateral thoracic paraspinal muscles in the VCP^R155H/R155H^ mice (average age 14–21 days of age), but not in the control WT animals. The VCP mutant mice depicted abnormal motor units, myotonic discharge, normal insertional activity, but no fibrillations and fasciculation potentials. A reduction in recruitment and interference patterns was also observed in these VCP^R155H/R155H^ mice (**Table 1**).

**Table 1:** EMG measurements of myopathic changes in VCP^R155H/R155H^ versus WT littermates.

*VCP^R155H/R155H^ versus WT Electrodiagnostic Measurements*	Insertional Activity	Fibrillations	Fascicu-lations	Amp	Recruitment and Interference
Thoracic paraspinal	Normal	None	None	Mixed	Reduced
Right tibialis anterior	Normal	None	None	Mixed	Reduced
Right hamstrings	Normal	None	None	Mixed	Reduced
Right medial gastrocnemius	Normal	None	None	Mixed	Reduced
Left tibialis anterior	Normal	None	None	Mixed	Reduced
Left hamstrings	Normal	None	None	Mixed	Reduced
Left medial gastrocnemius	Normal	None	None	Mixed	Reduced

Thoracic paraspinal, right tibialis anterior, right hamstrings, right medial gastrocnemius, left tibialis anterior, left hamstrings and left medial gastrocnemius muscle groups in Wild Type mice were all tested and found to be normal in all categories.

### Impaired autophagic pathway in the homozygote VCP^R155H/R155H^ quadriceps

**Figure 2:**
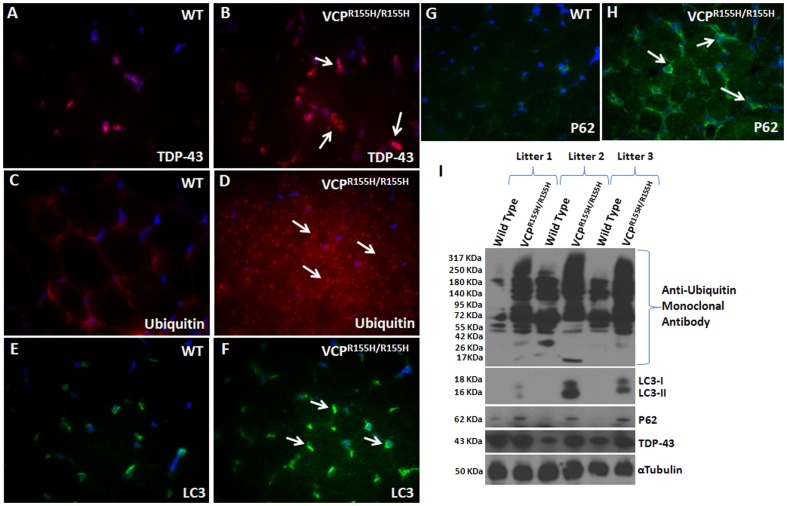
Immunohistochemical analysis of TDP-43 inclusions, ubiquitin, LC3-I/II, and P62 expression levels in VCP^R155H/R155H^ and WT quadriceps. Quadriceps sections revealed increased expression of (A–B) TDP-43, (C–D) ubiquitin, (E–F) LC3-I/II and (G–H) P62 antibodies in VCP^R155H/R155H^ mice as compared to their WT littermates (Mag 63×). (I) Western blot analyses confirmed the increased TDP-43, ubiquitin, LC3-I/II and P62 protein expression levels in the VCP^R155H/R155H^ mice. Alpha tubulin was used as a loading control.

The autophagic process involves the encapsulation of the constituents into autophagosomes, whereby fusion with lysosomes creates autolysosomes. Ubiquitination also serves as a signal for protein degradation by the 26S proteasome. To determine whether the autophagic pathway was disturbed in these 15-day old VCP^R155H/R155H^ and WT mice, we performed immunohistochemistry with TDP-43, ubiquitin, LC3-I/II and P62 autophagy cascade markers (**Figure 2**). As compared to the WT (**Figure 2A**), we discovered TDP-43 positive sarcoplasmic inclusions, most commonly in small angular muscle fibers in the VCP^R155H/R155H^ (**Figure 2B**). The myofibrils of the VCP^R155H/R155H^ mice contained ubiquitin aggregates and inclusions (**Figure 2C**) in comparison with their control WT littermates (**Figure 2D**). LC3, an autophagosome marker, was highly expressed in the VCP^R155H/R155H^ mutant mice (**Figure 2F**) when compared to the WT animals (**Figure 2E**). P62 immunoreactivity substrates for selective autophagy were detected in the cytoplasmic and nuclear areas of VCP^R155H/R155H^ quadriceps muscles versus WT mice (**Figure 2G,H**). Western blot analyses with these autophagy markers confirmed these findings (**Figure 2I**). VCP expression levels in the quadriceps of VCP^R155H/R155H^ homozygote and WT animals were comparable by immunohistochemical and Western blot analyses (**Figure S1 A–C**).

### Mitochondrial staining in quadriceps of VCP^R155H/R155H^ mice

**Figure 3:**
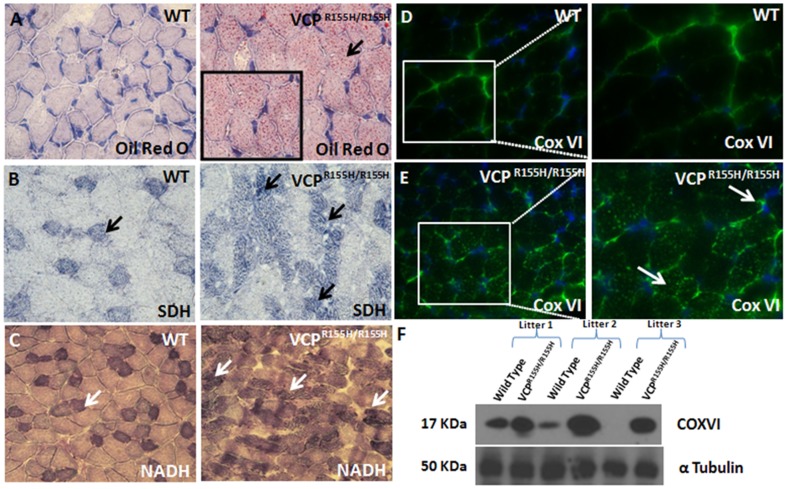
Immunohistochemical analysis of mitochondrial markers in VCP^R155H/R155H^ and WT quadriceps. (A) Oil Red O staining of WT and VCP^R155H/R155H^ mice quadriceps reveals increased lipid composition (as shown in inset) (Mag 63×). (B) Staining with SDH enzyme complex revealed deep-purple regions in Type 1 fibers, reflective of increased oxidative potential in these fibers and a scattered purple-speckled appearance in Type 2 fibers in the VCP^R155H/R155H^ and (C) increased NADH (reduced form production) in the VCP^R155H/R155H^ as compared to their WT controls (“checkered” pattern) (Mag 63×). Immunohistochemical analyses of COX-IV in (D) WT and (E) VCP^R155H/R155H^ mice. (F) Western blot analyses of COX-IV protein expression levels in the WT and VCP^R155H/R155H^ mice. Alpha tubulin was used as a loading control.

Identification of oxidative and non-oxidative fibers is an important property to visualize in assessing mitochondrial pathology and diseases. Thus, we determined the mitochondrial oxidative potential and proliferation by staining for Oil Red O to detect lipid composition and various mitochondrial complexes including COX-IV, succinic dehydrogenase (SDH) enzyme and its reduced form of nicotinamide adenine dinucleotide (NADH) in VCP^R155H/R155H^ and WT mice quadriceps. Staining revealed infiltration of triglycerides and lipids in the VCP^R155H/R155H^ quadriceps muscles fibers (inset) compared to their WT controls by Oil Red O (**Figure 3A**). Staining with the SDH enzyme complex revealed deep-purple regions in Type 1 fibers, reflective of increased oxidative potential in these fibers and a scattered purple-speckled appearance in Type 2 fibers (**Figure 3B**). Similarly, staining with NADH depicted an increased NADH production observed in the VCP^R155H/R155H^ as compared to their WT controls (“checkered” pattern) (**Figure 3C**). The SDH and NADH stains also revealed grouping of Type 1 fibers, suggestive of chronic neurogenic changes with reinnervation within the VCP^R155H/R155H^ fibers (**Figure 3B,C**). Immunohistochemical (**Figure 3D,E**) and Western blot analyses (**Figure 3F**) revealed increased respiratory chain enzyme COX-IV expression levels within the VCP^R155H/R155H^ quadriceps muscles when compared to age-matched WT littermates.

### Histological analyses in brains of VCP^R155H/R155H^ mice

**Figure 4:**
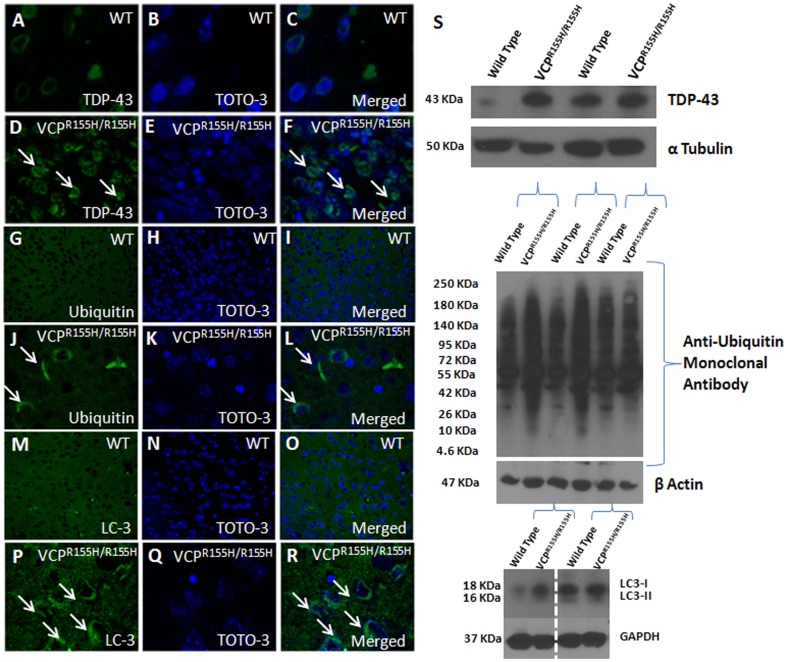
Immunohistochemical and Western blot analyses of TDP-43, ubiquitin, and LC3I/II expression in VCP^R155H/R155H^ and WT brains. TDP-43 expression levels in (A–C) WT and (D–F) VCP^R155H/R155H^ animals. Ubiquitin expression levels in (G–I) WT and (J–L) VCP^R155H/R155H^ animals. LC3I/II expression levels in (M–O) WT and (P–R) VCP^R155H/R155H^ animals. (S) Western blot analyses of TDP-43, ubiquitin and LC3I/II in brains of WT and VCP^R155H/R155H^ animals. Alpha tubulin, β actin, and GAPDH were used as loading controls.

Slight cytosolic accumulation or dense deposits of TDP-43 were observed in these mice, as TDP-43 expression was mostly localized in the nuclei of both VCP^R155H/R155H^ and WT mice (**Figure 4A–F**). Perinuclear and cytosolic accumulations of ubiquitin-positive deposits were observed in the VCP^R155H/R155H^ mice, but not in the WT mice (**Figure 4G–L**). In order to determine the role of autophagy in the 15-day old VCP^R155H/R155H^ mouse model brains, we stained with LC3-I/II-specific autophagy markers. There was slightly increased LC3-I/II immunoreactivity in the VCP^R155H/R155H^ than in the WT control littermates (**Figure 4M–R**). Western blot analyses of the VCP^R155H/R155H^ mice brains depicted increased levels of TDP-43, ubiquitin-positive aggregates, and LC3-I/II proteins (**Figure 4S**) compared with WT littermates. Evaluation of the VCP distribution pattern by IHC analyses demonstrated equal expression levels amongst the VCP^R155H/R155H^ and WT mice brains (**Figure S1 D–I**).

**Figure 5:**
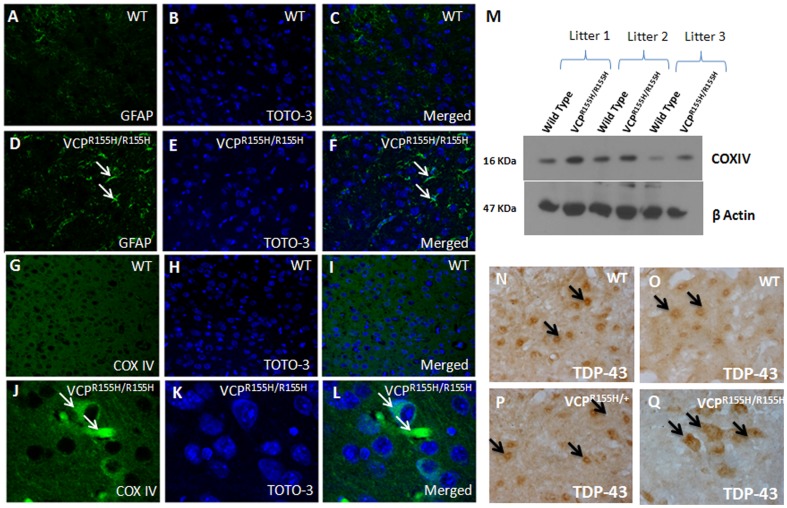
Immunohistochemical analyses of GFAP and COX-IV expression in VCP^R155H/R155H^ and WT brains and spinal cords. GFAP expression levels in (A–C) WT and (D–F) VCP^R155H/R155H^ animals. COX-IV enzyme expression levels in (G–I) WT and (J–L) VCP^R155H/R155H^ animals. (M) Western blot analysis of COX-IV enzyme expression level in VCP^R155H/R155H^ and WT brains. β actin was used as a loading control. (N–Q) TDP-43 immunoreactivity of the WT, VCP^R155H/+^, and VCP^R155H/R155H^ spinal cords. Note the nuclear and occasional faint cytosolic staining of TDP-43 in the (N,O) WT littermates in contrast to the strong cytosolic aggregation of TDP-43 seen in the (P) VCP^R155H/+^ heterozygote and (R) VCP^R155H/R155H^ homozygote mice (as indicated by black arrows) (Bar = 50 µm, Mag 40×).

To better understand the role of astrocyte mechanical strength maintenance, astrocytes were immunostained with GFAP. In comparison to the WT littermates (**Figure 5A–C**), an increase of GFAP staining in VCP^R155H/R155H^ mice was observed suggestive of gliosis (**Figure 5D–F**). We also evaluated mitochondrial pathology in the 15-day old VCP^R155H/R155H^ and WT brains (**Figure 5G–I**) and found an increased expression of COX-IV in the mutant mice (**Figure 5J–L**). Western blot analyses depicted an increased level of COX-IV expression in the VCP^R155H/R155H^ versus WT brains (**Figure 5M**).

### Rapid motor neuron degeneration in the VCP^R155H/R155H^ spinal cords

Mutations in *VCP* have been linked to 1–2% of familial ALS cases. Therefore, we examined the 10-day old VCP mouse spinal cords by staining with TDP-43 antibody. Compared with WT littermates (**Figure 5N,O**), marked MN degenerative changes were noted in the heterozygote VCP^R155H/+^ animals along with widespread occurrence of TDP-43 labeled cytosolic aggregates (**Figure 5P**). Homozygote VCP^R155H/R155H^ mice developed rapid MN degeneration with prominent TDP-43 pathology (**Figure 5Q**).

### Homozygote VCP^R155H/R155H^ microCT imaging reveals Paget-like bone lesions

**Figure 6:**
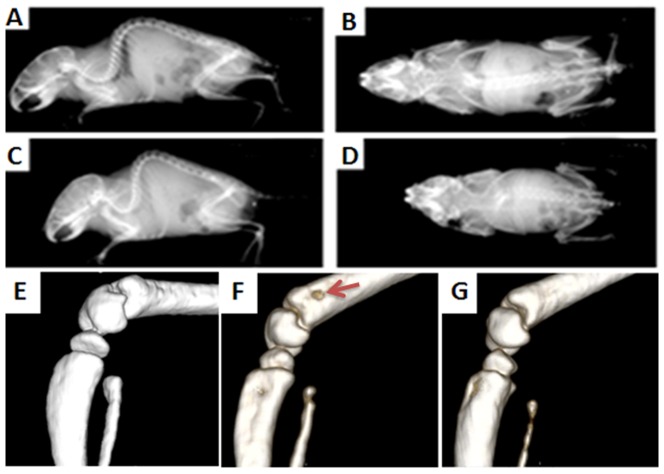
MicroCT and hind limb bone imaging in VCP^R155H/R155H^ and WT mice. MicroCT images in (A,B) WT and (C,D) homozygote VCP^R155H/R155H^ animals. Bone images of the (E) left hind limb bone in WT and (F,G) left and right hind limb bones in VCP^R155H/R155H^ animals showing a Paget-like lesion (shown by red arrow), respectively.

Paget disease of bone (PDB) is a common disorder characterized by increased turnover of bone within lesions. In order to investigate the lucencies and bone turnover, we performed microCT and bone imaging in the VCP^R155H/R155H^ and WT mice. MicroCT imaging analysis of the WT depicted normal CT scan (**Figure 6A,B**), whereas VCP^R155H/R155H^ mice revealed radiolucencies of the proximal tibiae (**Figure 6C,D**). As compared to the WT normal bones without any lesions (**Figure 6E**), bone analyses in the VCP^R155H/R155H^ mice depicted non-symmetrical Paget-like lesions of the right hind limb bone (**Figure 6F**), suggestive of PDB and not in the left hind limb bone (**Figure 6G**).

Collectively, the data show that the VCP^R155H/R155H^ mouse model has the features of VCP-associated human disease, demonstrating severe muscle weakness, centrally localized nuclei in muscle, mitochondrial pathology in muscle, heart and brain, Paget-like lesions, motor neuron degeneration, and increased expression levels of the TDP-43, ubiquitin-positive aggregates and autophagy markers.

## Discussion

Despite intense research efforts, VCP-associated disease and ALS remain fatal degenerative neuromuscular disorders with poorly understood pathogenesis and no effective treatment. 90% of patients develop muscle weakness at a mean age of 42 years of age, 51% of patients developing PDB, and 32% of patients developing FTD [1]. Patients with this disease depict degenerating fibers, rimmed vacuoles, and ubiquitin- and TDP-43-positive inclusions. Recently, mutations in the *VCP* gene have been linked with 2% of familial ALS [11] with patients demonstrating neurodegenerative changes and asymmetric focal weakness of the extremities or bulbar findings. VCP is highly conserved in evolution, plays a critical role in a plethora of cellular functions and is involved in several signaling transduction pathways. The present investigation explores and characterizes the first homozygote VCP^R155H/R155H^ mouse model as an accelerated model of VCP-associated neurodegenerative diseases.

Previous studies have reported the VCP knockout mouse models which was lethal whereas the hemizygote mice were indistinguishable from their WT littermates [37]. Over the years, mouse models have been generated either overexpressing the mutant VCP allele under a muscle-specific promoter revealing muscle weakness and vacuoles [38] or under a ubiquitous promoter recapitulating the full spectrum of VCP disease, including muscle weakness, pathology of inclusion body myopathy with rimmed vacuoles, and TDP-43 pathology [39]. Our laboratory has previously generated a heterozygote knock-in VCP^R155H/+^ mouse model [34], with relatively mild progressive muscle weakness, microCT evidence of Paget-like lesions at the ends of long bones and progressive cytoplasmic accumulation of TDP-43, ubiquitin-positive inclusion bodies and increased LC3-II staining in the quadriceps, brain and spinal cord pathology of the motor neurons cells, suggestive of ALS. Previous ALS mouse models include ones involving the SOD1, TDP-43 as well as the DNA/RNA binding proteins FUS (fused in sarcoma) genes [40], [41].

This is the first investigation to report the VCP^R155H/R155H^ mouse model as an accelerated system for human VCP-associated diseases. The homozygote VCP^R155H/R155H^ mice remained significantly smaller and lethality was observed by 14–21 days of age. Characterization of these mice revealed accelerated weakness, muscle, spinal cord, brain and cardiac pathology, abnormal skeletal architecture, pathological muscle mitochondrial structure, and brain degeneration. Autophagy plays a balancing role in the self-degradative process, it is important in response to nutrient stress and in clearing damaged organelles and intracellular pathogens. Autophagic degradation is involved in Alzheimer and Huntington's diseases, among other neurodegenerative diseases, and in inflammatory disorders [42]. Mutations in p62/SQSTM1, a multifunctional adaptor protein, are responsible for approximately 10% of sporadic PDB, 50% of familial PDB cases and most recently mutations have been associated with ALS [43], [44], [45], [46]. In our VCP mouse model, accumulation of P62 and LC3-I/II markers suggests impaired autophagy. Our results thus offer further insight on the role of VCP and autophagy in motor neuron biology. ALS is a neurodegenerative disease involving both upper and lower motor neurons, and is caused by *VCP* gene mutations in up to 2–3% of isolated familial ALS [11]. We examined the VCP^R155H/R155H^ mouse spinal cords and discovered significant motor neuron degeneration and TDP-43-positive cytoplasmic accumulation, associated with ALS pathology.

The precise mechanistic fashion by which the VCP R155H mutation and other mutations are associated with neurodegenerative diseases has not yet been fully established. Preliminary evidence suggests VCP mutations are implicated not only in the disrupted proteasome/ubiquitin, but also in the autophagy/mitophagy pathways [47], [48], [49]. Mitochondria are the major organelles providing the energy, thus maintaining a healthy source of mitochondria is crucial to overall cell fitness for many cellular functions. However, mitochondria are a major source of reactive oxygen species (ROS), consuming cytosolic ATP when dysfunctional. Several studies have placed an emphasis on the mitochondria and its interaction between cellular death, autophagy and inflammation signaling pathways [50]. Evidence suggests that VCP may play an important role in maintaining an active mitophagic process. A recent study has shown the importance of the ubiquitination of mitofusins Mfn1 and Mfn2 (2 large GTPases which mediate fusion of mitochondria) which mediate mitophagy and leads to their degradation by the proteasome and p97 [51]. EM analyses of the VCP^R155H/R155H^ quadriceps and brain depicted abnormal mitochondrial proliferation and aberrant sarcomeric architecture with large megaconia and disrupted cristae. Lipid accumulation and increased SDH intensity in the Type 1 fibers and increased NADH reaction product in the VCP^R155H/R155H^ mutant mice are suggestive of increased oxidative fibers and higher mitochondrial density in these muscle tissues. Further functional mechanistic studies are needed to explore the intricate relationship between VCP and its substrates, as well as its interactions with ubiquitin intermediates, autophagosome-associated proteins, mitochondria and the various disrupted signaling transduction cascades in the etiology of this and other neurodegenerative diseases.

### Conclusions

Currently, there are no effective therapies for IBMPFD and ALS, thus, *in vivo* and *in vitro* studies utilizing the VCP^R155H/R155H^ experimental mouse model will provide an excellent platform for translational studies to discover novel treatments for patients with progressive neurodegenerative diseases.

## Supporting Information

Figure S1 **Immunohistochemical and Western blot analysis of VCP expression levels in VCP^R155H/R155H^ and WT animals.** IHC analysis of VCP distribution in quadricep muscles of (A) WT and (B) VCP^R155H/R155H^ mice. (C) Western blot analyses depicting equal VCP expression levels in quadriceps muscles of WT and VCP^R155H/R155H^. Alpha tubulin was used as a loading control. IHC analysis of VCP distribution in brains of (D–F) WT and (G–I) VCP^R155H/R155H^ mice (as shown by arrows).
(TIF)
