# Supplementary figures and images for: The Homozygote VCPR155H/R155H Mouse Model Exhibits Accelerated Human VCP-Associated Disease Pathology
Source: PLoS One. 2012 Sep 28;7(9):e46308. doi: 10.1371/journal.pone.0046308 (PMC3460820; doi:10.1371/journal.pone.0046308)

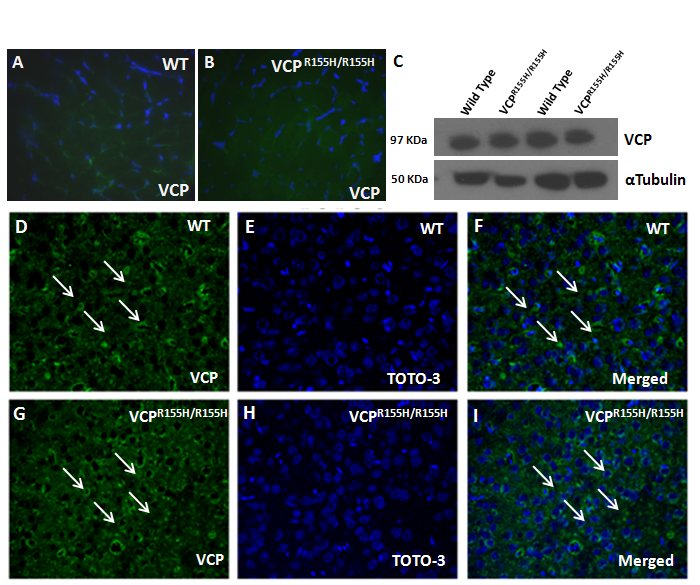

Supplement: Figure S1 — Immunohistochemical and Western blot analysis of VCP expression levels in VCPR155H/R155H and WT animals. IHC analysis of VCP distribution in quadricep muscles of (A) WT and (B) VCPR155H/R155H mice. (C) Western blot analyses depicting equal VCP expression levels in quadriceps muscles of WT and VCPR155H/R155H. Alpha tubulin was used as a loading control. IHC analysis of VCP distribution in brains of (D–F) WT and (G–I) VCPR155H/R155H mice (as shown by arrows). (TIF) [file pone.0046308.s001.tif]
